# Complete genome sequence of *Metarhizium marquandii* strain ISA501

**DOI:** 10.1128/mra.00106-25

**Published:** 2025-06-20

**Authors:** Jun Zhou, Maria Elena Antinori, Gabriele Bellotti, Edoardo Puglisi, Michel Chalot

**Affiliations:** 1 Chrono-environnement (UMR 6249), Université Marie et Louis Pasteur, CNRS 129611 , Montbéliard, France; 2 Department for Sustainable Food Process, Università Cattolica del Sacro Cuore 9371, Piacenza, Italy; 3 ISA Innovations for Sustainable Agriculture R&D, srl, Piacenza, Italy; University of Maryland School of Medicine, Baltimore, Maryland, USA

**Keywords:** *Metarhizium marquandii*, genome, rhizosphere

## Abstract

We report the complete genome sequence of *Metarhizium marquandii* strain ISA501, isolated from maize rhizosphere soil. The 42.8 Mb genome, assembled with hybrid sequencing, comprises eight chromosomes with 51.65% GC content. This high-quality resource provides insights into its plant growth-promoting potential, secondary metabolism, and ecological roles in sustainable agriculture.

## ANNOUNCEMENT

The genus *Metarhizium* comprises fungi known for their entomopathogenic properties and use as biocontrol agents (1). Some species also promote plant growth by associating with roots (2). Among them, *Metarhizium marquandii* has been reported as a saprophyte with plant growth-promoting abilities and roles in improving soil health (3, 4). However, no genome sequence has been made publicly available to date. Here, we report the chromosome-level genome assembly of strain ISA501, isolated from maize rhizosphere soil in Brescia, Italy (N 45.333096, E 10.438125).

**Fig 1:**
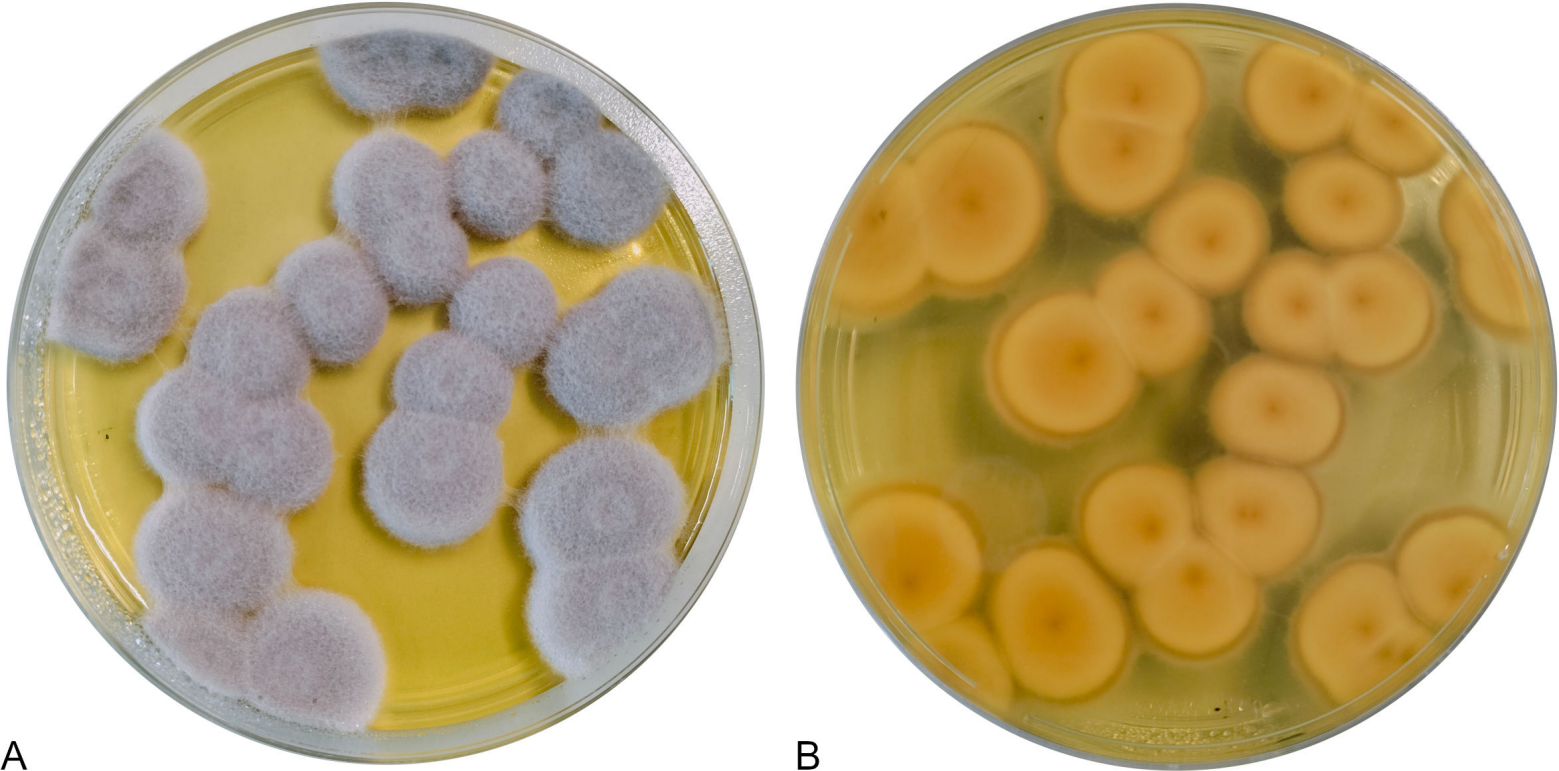
*Metharizium marquandii* ISA501 grown 10 days on PDA. (A) Upper side of the plate. (B) Bottom of the plate. The medium becomes yellowish, as in the picture, as soon as the strain starts growing, probably due to the production of some metabolites.

Isolation was performed using a modified protocol based on Barillot et al. (5). Rhizospheric soil was suspended in 0.9% NaCl and agitated for 1 hour. Dilutions were plated on PDA with 0.1 mg/mL chloramphenicol. Colonies were transferred to antibiotic-free PDA, and single spores were isolated. Cultures were grown at 28°C for 7 days before DNA extraction (Fig. 1). Species identity was initially determined by ITS sequencing (primers ITS1 and ITS4) (6), followed by BLAST comparison against GenBank. The top hit was *M. marquandii* CBS 182.27 (NR_131994). For genome sequencing, fungal mycelia were grown in potato dextrose broth at 28°C for 7 days, harvested, washed, freeze-dried, and used for genomic DNA extraction (HiPure Universal DNA Kit, Genepioneer Biotechnologies).

A hybrid sequencing strategy was employed to ensure the accuracy and completeness of the genome assembly. DNA libraries for short-read sequencing were constructed using the VAHTS Universal DNA Library Prep Kit for Illumina V3 ND607 (Vazyme). Sequencing was conducted on the Illumina NovaSeq 6000 platform in paired-end 150 bp mode, generating 21,053,627 reads, amounting to 6.32 Gb of data, with an average depth of coverage of approximately 144×. For Nanopore sequencing, DNA was purified using 0.4× magnetic beads to remove fragments <2 kb. Long-read sequencing libraries were prepared using the SQK-LSK110 ligation kit (Nanopore) and sequenced on the PromethION 48 platform (R9.4.3 flow cells). This yielded 992,019 reads with a total output of 9.94 Gb, achieving an average coverage depth of 214×. The *N*_50_ read length was 18,680 bp. Guppy (high-accuracy mode) was used for basecalling. Quality control for both data sets involved removing low-quality reads and adapters using fastp (version 0.20.0) (7) and Filtlong (version 0.2.1). Genome assembly was performed by integrating short and long reads. Nanopore reads were corrected using Canu (version 2.1.1) (8) and LoRDEC (version 0.9) (9). Primary assembly was achieved using NextDenovo (version 2.5) (10), and potential misassemblies were resolved by aligning corrected reads to the draft assembly with minimap2 (version 2.1) (11). Final polishing was conducted using three iterations of NextPolish (version 1.4.0) (12) with Nanopore reads and Pilon (version 1.23) (13) with Illumina reads. Default parameters were used except where otherwise noted.

The final genome assembly comprises eight chromosomes, totaling 42,805,715 bp, with a GC content of 51.65%. The largest chromosome is 7,777,640 bp. The *N*_50_ value is 7,303,840 bp. Completeness was assessed using BUSCO (version 5.2.1) (14), revealing 98.0% complete BUSCOs (96.7% single copy and 1.3% duplicated), with 0.3% fragmented and 1.7% missing (Table 1). Coverage analysis with minimap2 confirmed even read distribution. Telomeric repeats (CCCTAA)n were detected at both ends of each contig, supporting telomere-to-telomere completeness.

**TABLE 1:** Genome sequencing statistics

Parameter	Value
Illumina sequencing	
Average depth of coverage, Illumina short reads	~144×
Number of reads	21,053,627
Total bases (bp)	6,316,088,100
Nanopore sequencing	
Average depth of coverage, ONT long reads	~214×
Number of reads	992,019
Total bases (bp)	9,944,606,976
*N*_50_ read length (bp)	18,680
Genome assembly	
No. of chromosomes	8
Total length (bp)	42,805,715
Largest chromosome	7,777,640
GC (%)	51.65
*N*_50_ (bp)	7,303,840
*L*_50_	3
Genome assembly assessment	
Complete BUSCOs	98.0%
Complete and single-copy BUSCOs	96.7%
Complete and duplicated BUSCOs	1.3%
Fragmented BUSCOs	0.3%
Missing BUSCOs	1.7%

## Data Availability

The genome and sequencing data have been deposited at NCBI under BioProject accession PRJNA1215054. The BioSample accession is SAMN46395144. The Illumina sequencing reads are available in the NCBI Sequence Read Archive under accession SRR32309563, while the nanopore sequencing reads are accessible under accession SRR32316793. This Whole Genome Shotgun project has been deposited in DDBJ/ENA/GenBank under the accession no. JBLFEU000000000. The version described in this paper is the first version, JBLFEU010000000.
